# *In Vivo* Evaluation of the Antiasthmatic, Antitussive, and Expectorant Activities and Chemical Components of Three *Elaeagnus* Leaves

**DOI:** 10.1155/2015/428208

**Published:** 2015-10-21

**Authors:** Yuebin Ge, Fei Zhang, Qin Qin, Yingying Shang, Dingrong Wan

**Affiliations:** School of Pharmacy, South-Central University for Nationalities, Wuhan 430074, China

## Abstract

The leaf of *Elaeagnus lanceolata* and *Elaeagnus henryi* as well as *Elaeagnus pungens* has been documented as an effective herb for the treatment of asthma and chronic bronchitis in traditional clinical medicine. This study was aimed at evaluating the antiasthmatic, antitussive, and expectorant activities of the water extracts from the three plants *in vivo *and analyzing their chemical components by HPLC-DAD. At the medium and high doses, the water extracts of three *Elaeagnus *leaves significantly prolonged the preconvulsive time (*P* < 0.01) in guinea pigs, lengthened the latent period of cough (*P* < 0.01) and decreased the cough frequency caused by aqueous ammonia in mice (*P* < 0.01), and enhanced tracheal phenol red output in mice (*P* < 0.01). There were no significant differences in the pharmacological actions between the three *Elaeagnus* leaves. Moreover, there was more similarity on overlap peaks in the range of retention time from 10 to 40 min by HPLC and many peaks that belonged to flavonoids compounds. It suggested that the main constituents of the three *Elaeagnus *leaves were flavonoid for the pharmacological activities. These effects were the important evidence for the traditional use of *E. henryi* leaf and *E. lanceolata *leaf as well as* E. pungens *to treat asthma and chronic bronchitis.

## 1. Introduction

Asthma and chronic bronchitis are the chronic inflammatory diseases of the respiratory tract, which are characterized by increased airway hyperresponsiveness and mucus production that leads to episodes of wheezing, coughing, and shortness of breath [1]. Current pharmacological management of these diseases is mainly based on corticoids as anti-inflammatory agents in combination with *β*_2_-adrenergic agonists as bronchodilators [2]. However, these drugs will cause serious side effects. Asthma and chronic bronchitis have a worldwide incidence of 10% in adults and 35% in children. So, the high incidence of the diseases among the individuals presents that research on medications for the repetitious chronic diseases is very important. Particularly, it will be an alternative path to search for effective medicines in the rich traditional medicine in the world [3–5].

The family Elaeagnaceae consists of three genera including* Hippophae*,* Elaeagnus, *and* Shepherdia*. There are about 80 species of* Elaeagnus*, which are widespread in subtropical and temperate areas of East and Southeast Asia.* Elaeagnus pungens* leaf has been documented in the early traditional Chinese medicine (TCM) material medica “Bencao Gangmu” (Ming dynasty, about 430 years ago). It is documented as an antiasthmatic remedy to treat severe asthma, cough, bronchitis, or other respiratory disorders. According to the theory of traditional Chinese herbology in the early material medica “Zhongzang Jing,”* Elaeagnus pungens* leaf belongs to the herb of moisturizing lung and cough relieving, and it can astringe the dissipated lung Qi in individuals owing to the acerbity-astringent nature and flavour of the herb.

Through the resource and medical value survey of the* Elaeagnus* plants in minor nationality areas including Hubei province, Tujia nationality, and Guizhou and Yunnan provinces, we found that* Elaeagnus henryi* Warb. ex Diels. and* Elaeagnus lanceolata* Warb. are also generally used as* Elaeagnus pungens* to treat shortness of breath, cough, or bronchitis. On the clinical application, the leaf of the three* Elaeagnus* plants is decocted with water, grinded for powder, or prepared for Chinese patent medicines. Previous researches have shown that the plants from* Elaeagnus* contain some chemical constituents including flavonoid, lignanoids, organic acids, and terpenoids and have pharmacological effects such as antinociceptive, anti-inflammatory, and cytotoxic actions [6–16]. They have also been verified as nontoxic under oral administration for a long time in adult and in mice [17].

About* E. pungens* leaf, our previous study focuses on the chemical constituents [18, 19], content determination [20], the antiasthmatic, antitussive, and expectorant activities* in vivo* [21], and the relaxant mechanism* in vitro* [22]. Also, we studied the microscopic characteristics of the powders of* E. pungens*,* E. henryi,* and* E. lanceolata *and identified them with infrared spectroscopy [23]. However, whether the leaves of* E. henryi* and* E. lanceolata* have the antiasthmatic, antitussive, and expectorant activities as well as* E. pungens* in animals is still unknown. Furthermore, the relation between the chemical constituents and the active pharmacology effects of the* Elaeagnus* plants has not been investigated. The present study was purposed to compare the activities of the water extracts of three* Elaeagnus* leaves in terms of the antiasthma, antitussive, and expectorant effect* in vivo*. And the chemical components of them were also analyzed by HPLC-DAD. The evaluation will serve as the basis for further research on resources and medical application of the* Elaeagnus *plants.

## 2. Materials and Methods

### 2.1. Collection and Preparation of Plant Material

The fresh leaves of* Elaeagnus pungens*,* Elaeagnus lanceolata,* and* Elaeagnus henryi* were collected in October 2014 at Huangmei, Badong, Jianshi, Hubei province, China, respectively. The plants were authenticated by Dr. Dingrong Wan, Professor in Pharmacognosy at School of Pharmacy, South-Central University for Nationalities, with the voucher specimen numbers SCUN 1208002, SCUN 1208004, and SCUN 1208008, respectively. The collected leaves were dried in shade and reduced to coarse powder using a mortar and pestle.

### 2.2. Extract Preparation

The dried leaves of three* Elaeagnus* plants were extracted with water two times. It was decocted for 1 h each time. The combined solution was filtered and concentrated under reduced pressure to afford the water extract. The yields of the water extract were expressed as the weight percentage of obtained extract in the total weight of crude material, specifically, 22.6%, 17.9%, and 16.9% for* E. pungens*,* E. lanceolata,* and* E. henryi*, respectively.

### 2.3. Animals and Administration

Guinea pigs of either sex (150–200 g) for antiasthmatic experiments and Kunming mice of either sex (22–25 g) for antitussive and expectorant experiment were purchased from Hubei Province Center for Disease Control and Prevention (Wuhan, China). All animals were housed at room temperature (22–24°C) and constant humidity (50–60%) under a 12 h light-dark cycle in SPF grade laboratory. The animal study was performed according to the international rules considering animal experiments and the internationally accepted ethical principles for laboratory animal use and care.

After 3–5 days of adaptation, the eligible animals were randomly assigned to eleven groups and orally administered, including control group (distilled water), positive group (aminophylline/125 mg/kg, pentoxyverine/50 mg/kg, or ammonium chloride/1000 mg/kg for antiasthmatic, antitussive, or expectorant experiment, resp.), and the water extract groups (low, medium, and high doses). In the tests, administrated dose were 2.7, 5.4, and 10.8 g/kg for guinea pigs and 4.4, 8.8, and 17.6 g/kg for mice (expressed as being equal to the weight of crude material per body weight), which were calculated by coefficient commutation of somatotypes and yield of extract (the used dosage was the medium dose, being five times by clinical dosage of 15 g crude herb in adults). After treatment for 5–7 days, activities were tested and evaluated.

### 2.4. The Antiasthmatic Test* In Vivo*

To screen the sensitivity, guinea pigs were placed in a glass chamber (3 L) and sprayed with the mixture of 0.1% histamine and 2% acetylcholine chloride (1 : 1, v/v) under the average pressure of 450 ± 50 mmHg for 15 s [24]. The times to onset of respiratory distress and tumble (preconvulsive time) were recorded. The guinea pigs with preconvulsive time of more than 120 s were considered to be insensitive and discarded. The eligible guinea pigs were randomly allotted to groups (*n* = 8) and administered according to Section 2.3 for 5 days. The administration on day 5 was given at 1.5 h before the measurement of preconvulsive time. The delitescence of convulsion and tumble for each guinea pig within 6 min were observed.

### 2.5. Antitussive Test* In Vivo*

To screen the sensitivity, mice were placed in a glass chamber (0.5 L) and sprayed with 25% aqueous ammonia (w/v) under the average pressure of 400 ± 50 mmHg for 5 s. The mice were randomly allotted to eight groups (*n* = 10) and administered according to Section 2.3. All groups were treated with a single dose daily for 7 days and the last dose was given 1.5 h before the measurement of latent period of cough (from the start to the onset of cough) and frequency of cough. The frequency of cough was observed for 2 min.

### 2.6. Expectorant Test* In Vivo*

The procedures were performed as described previously [25]. Male and female mice were randomly allotted to eight groups (*n* = 10) and administered according to Section 2.3. All groups were treated with a single dose daily for 5 days and the last dose was given 1 h before intraperitoneal injection of phenol red solution (5% in saline solution, w/v, 0.1 mL/10 g body weight). Then 30 min after application of phenol red, the mice were anesthetized with pentobarbital at the dose of 75 mg/kg body weight and exsanguinated by cutting the abdominal aorta. After being dissected free from adjacent organs, the trachea was removed from the thyroid cartilage to the main stem bronchi and put into 1 mL normal saline immediately. After ultrasonic for 15 min, 1 mL NaHCO_3_ solution (5%, w/v) was added to the saline and optical density of the mixture was measured at 558 nm using WFZ UV-2000 UV–vis spectrophotometer (Shanghai Spectrum Instrument Co., Ltd., China).

### 2.7. Chemical Analysis by HPLC/DAD

About 1.0 g of three* Elaeagnus* leaves was immersed in water and decocted for 1 h. The extracted solution was filtered for the tested sample and detected by HPLC-DAD (Model 1200, Agilent Technologies Co. Ltd., USA). An aliquot of the filtrate (20 *μ*L) was injected into a Thermo ODS HYPERSIL C18 column (250 × 4.6 mm i.d., 5 *μ*m) and eluted with a linear gradient with a mobile phase containing solvent A (acetonitrile) and solvent B (0.1% phosphoric acid). The gradient elution program was 5–11% A in 0–5 min, 11–25% A in 5–30 min, 25–36% A in 40–60 min, and 36–80% A in 40–60 min. The flow rate was 1.0 mL/min, the effluent was monitored at 315 nm, and column temperature was set at 30°C. The monomeric compounds of C1 (kaempferol 3-*O*-*α*-rhamnopyranosyl-(1→2)[*α*-rhamnopyranosyl-(1→6)]-*β*-D-galactopyranoside), C2 (kaempferol 3-*O*-*α*-L-rhamnopyranosyl-(1→2)-*β*-D-glucopyranoside), and C3 (kaempferol 3-*O*-(6′′-*O*-*E*-*p*-coumaryl)-*β*-D-glucopyranoside) were also injected as the standard substances.

### 2.8. Statistical Analysis

Data obtained in all experiments was expressed as mean ± SD. Statistical analysis was done by one-way analysis of variance (ANOVA) with Tukey test using the software of Origin 7.0. Differences between means of treated groups and the control were regarded as significant at *P* < 0.05.

## 3. Results

### 3.1. Antiasthmatic Effects

**Figure 1:**
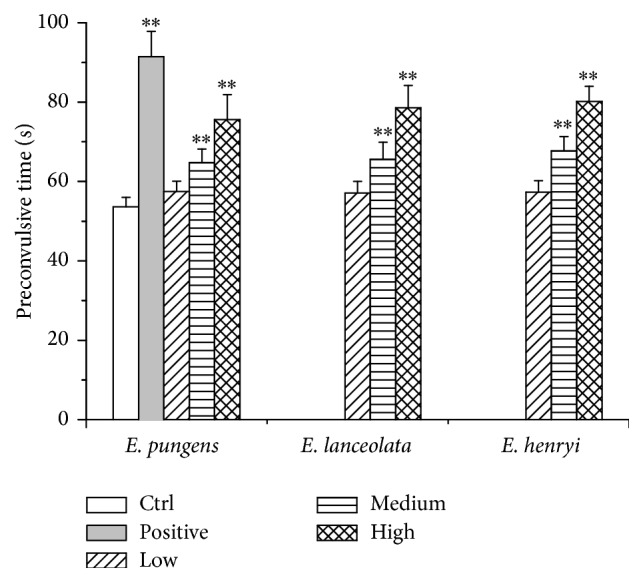
Effect of control, positive (aminophylline 125 mg/kg), water extracts of* E. pungens*,* E. lanceolata,* and* E. henryi *leaf (low, medium, and high doses of 2.7, 5.4, and 10.8 g/kg, expressed as being equal to the weight of crude material per body weight) on guinea pigs bronchoconstriction induced by mixture spraying histamine and acetylcholine chloride after administration for 5 days (*n* = 8). Values are presented as mean ± SD, ^*∗∗*^ *P* < 0.01, compared with control group.

The effects of the water extracts from three* Elaeagnus* leaves in guinea pigs exposed to mixture spray of 0.1% histamine and 2% acetylcholine chloride were shown in Figure 1. The preconvulsive times of eleven groups had no difference before administration (*P* > 0.05). After administration, the preconvulsive times were 53.6 ± 2.4, 91.5 ± 6.3, 57.5 ± 2.6, 64.8 ± 3.4, 75.6 ± 6.3, 57.1 ± 2.9, 65.6 ± 4.3, 78.6 ± 5.6, 57.3 ± 2.9, 67.7 ± 3.6, and 80.2 ± 3.8 s in the control, aminophylline, and low, medium, and high dose of the water extracts from the leaves of* E. pungens*,* E. lanceolata,* and* E. henryi *groups, respectively. It showed that aminophylline and medium and high dose of water extracts from the three* Elaeagnus* leaves increased the preconvulsive time by 70.71%, 20.90%, 41.05%, 22.39%, 46.64%, 26.31%, and 49.63%, respectively. Comparing with control group, there were very significant differences in aminophylline group and medium and high dose groups of tested extracts (*P* < 0.01) by ANOVA among all the groups.

### 3.2. Antitussive Effects

**Figure 2:**
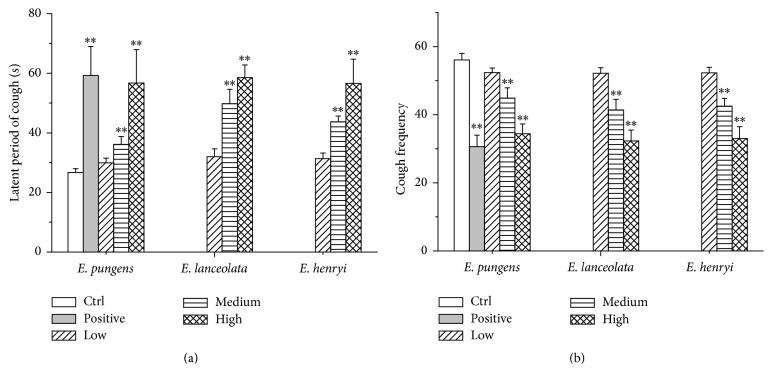
Effect of control, positive (pentoxyverine 50 mg/kg), water extracts of* E. pungens*,* E. lanceolata,* and* E. henryi *leaf (low, medium, and high doses of 4.4, 8.8, and 17.6 expressed as being equal to the weight of crude material per body weight) on the aqueous ammonia-induced latent period of cough (a) and cough frequency (b) after administration for 7 days (*n* = 10). Values are presented as mean ± SD, ^*∗∗*^ *P* < 0.01, compared with control group.

The antitussive effects of the water extracts from three* Elaeagnus* leaves on mice were shown in Figures 2(a) and 2(b). All water extracts induced latent period of cough and reduced cough frequency in a dose-dependent manner. Comparing with control group, there were significant differences in positive and medium and high dose groups of tested extracts (*P* < 0.01). The percentage of latent period of cough increased 121.72% (pentoxyverine), 35.21%, 112.36% (*E. pungens*), 86.52%, 119.10% (*E. lanceolata*), and 63.67%, 111.99% (*E. henryi*), respectively. And cough frequency was inhibited by 45.45% (pentoxyverine), 19.96%, 38.68% (*E. pungens*), 24.24%, 41.18% (*E. henryi*), and 26.2%, 42.42% (*E. henryi*), respectively.

### 3.3. Expectorant Effects

**Figure 3:**
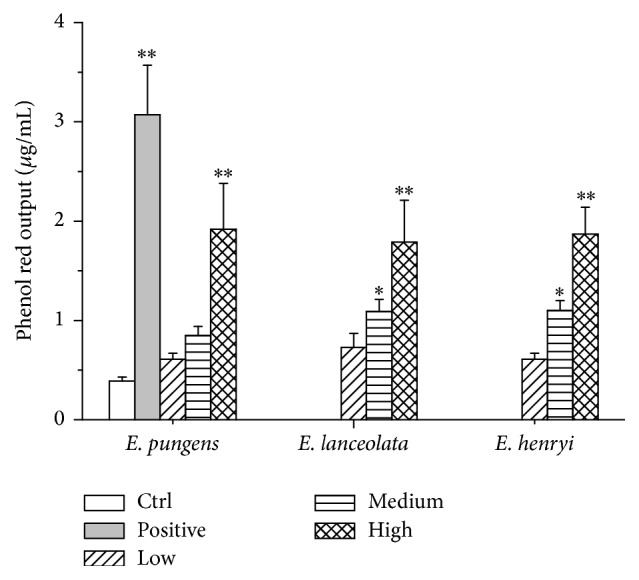
Effect of control, positive (ammonium chloride 1000 mg/kg), water extracts of* E. pungens*,* E. lanceolata,* and* E. henryi *leaf (low, medium, and high doses of 4.4, 8.8, and 17.6 expressed as being equal to the weight of crude material per body weight) on the volume of phenol red in mice's tracheas after administration for 5 days (*n* = 10). Values are presented as mean ± SD, ^*∗*^ *P* < 0.05, ^*∗∗*^ *P* < 0.01, compared with control group.

As shown in Figure 3, both positive and other tested groups prompted tracheal phenol red output after administration. It presented that the tracheal phenol red output in ammonium chloride and medium and high dose of water extracts from the leaves of* E. pungens*,* E. lanceolata,* and* E. henryi *groups increased by 6.87, 0.56, 1.18, 3.92, 0.87, 1.79, 3.59, 0.56, 1.82, and 3.79 folders, respectively. Except the low doses of three extracts and medium dose of* E. pungens*, the differences showed significantly in medium doses of other two plants (*P* < 0.05) and high doses of three plants (*P* < 0.01).

### 3.4. Chemical Analysis

**Figure 4:**
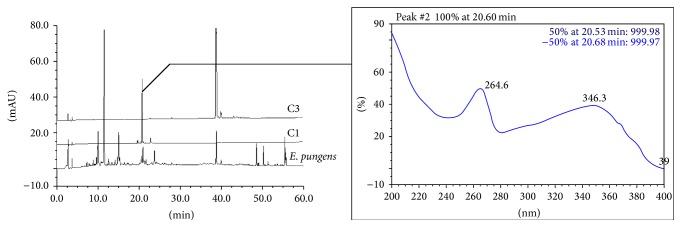
HPLC-DAD chromatographies of* E. pungens *leaf and monomeric compounds C1 (kaempferol 3-*O*-*α*-rhamnopyranosyl-(1→2)[*α*-rhamnopyran-osyl-(1→6)]-*β*-D-galactopyranoside, *t*_*R*_ = 20.600 min), C3 (kaempferol 3-*O*-(6′′-*O*-*E*-*p*-coumaryl)-*β*-D-glucopyranoside, *t*_*R*_ = 38.763 min).

**Figure 5:**
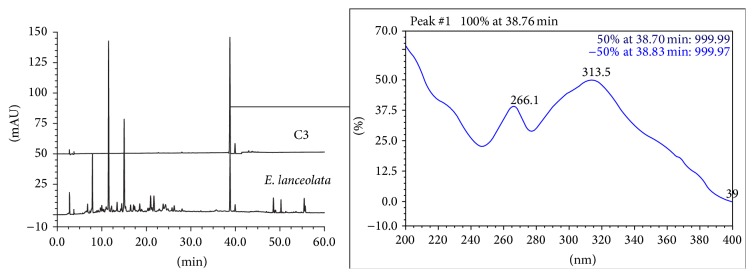
HPLC-DAD chromatographies of* E. lanceolata *leaf and monomeric compounds C3.

**Figure 6:**
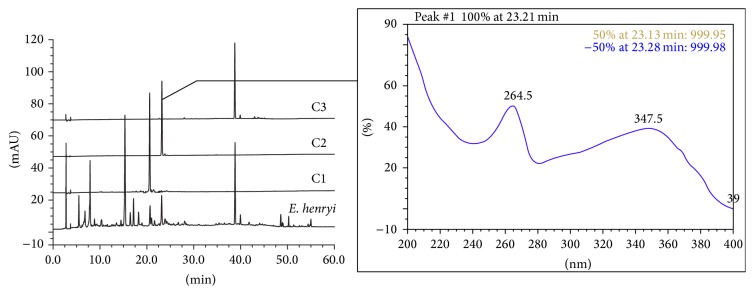
HPLC-DAD chromatographies of* E. henryi *leaf and monomeric compounds C1, C2 (kaempferol 3-*O*-*α*-L-rhamnopyranosyl-(1→2)-*β*-D-glucopyranoside, *t*_*R*_ = 23.100 min), and C3.

**Figure 7:**
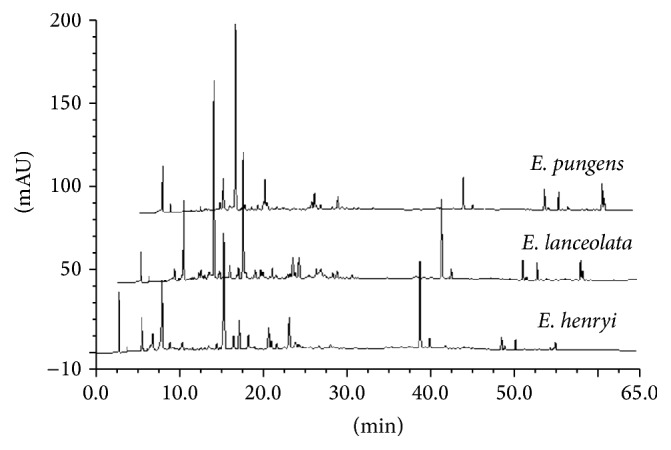
HPLC chromatographies of* E. pungens, E. lanceolata, *and* E. henryi *leaf.

The chromatographies of* E. pungens*,* E. lanceolata,* and* E. henryi *leaves by HPLC were shown in Figures 4–6. We found that the peaks at the retention time (*t*_*R*_) of 20.650 min and 38.733 min in the* E. pungens *leaf (Figure 4) were consistent with the monomeric compounds of C1 (kaempferol 3-*O*-*α*-rhamnopyranosyl-(1→2)[*α*-rhamnopyranosyl-(1→6)]-*β*-D-galactopyranoside, *t*_*R*_ = 20.600 min) and C3 (kaempferol 3-*O*-(6′′-*O*-*E*-*p*-coumaryl)-*β*-D-glucopyranoside, *t*_*R*_ = 38.763 min), respectively. Compared to the monomeric compounds, the overlap ratio of the absorptive curves by DAD was up to 0.97~0.99. In Figure 5, the overlap peak was *t*_*R*_ 38.797 min (C3) in the* E. lanceolata* leaf and three overlaps peaks was *t*_*R*_ 20.683 min (C1), 23.157 min (C2, kaempferol 3-*O*-*α*-L-rhamnopyranosyl-(1→2)-*β*-D-glucopyranoside, *t*_*R*_ = 23.100 min), and 38.87 min (C3) in the* E. henryi *leaf (Figure 6). Moreover, there was more similarity on overlap peaks between 10 and 40 min (Figure 7) and the peaks belonging to flavonoids compounds and characteristic absorptive wavelengths were also listed in Table 1.

## 4. Discussions

*Elaeagnus pungens* leaf has been traditionally used as an antiasthmatic remedy for several hundred years. It is nontoxic under oral administration for a long time in adult without definite IC_50_ value in mice [17]. Through the resource and medical value survey of the* Elaeagnus* plants in Hubei province, Tujia nationality area, and Guizhou and Yunnan provinces, we found that* Elaeagnus henryi* and* Elaeagnus lanceolata *are also generally used as* E. pungens* to treat shortness of breath, cough, or bronchitis. In the present study, water was chosen to extract the three* Elaeagnus* leavesaccording to the previous study suggesting that water fraction is the most active part of* E. pungens* leaf by the pharmacological evaluation [20]. Then, the water extracts of the three* Elaeagnus* leaves were evaluated on the relaxant, antitussive, and expectorant effects* in vivo*.

On the histamine and acetylcholine chloride-induced bronchoconstriction in guinea pigs, the water extracts significantly increased the preconvulsive time in asthma relieving (Figure 1). They also showed significant antitussive effect through the increase of latent period of cough and inhibition of cough (Figure 2). Additionally, the water extracts enhanced phenol red secretion into the airway with ammonium chloride as positive expectorant* in vivo* (Figure 3), which indicated that the expectorant action may be related to its ability to increase tracheobronchial mucus secretion and, thus, may decrease viscosity of mucus [26]. All three water extracts appeared to be the dose-dependent activities and significant differences at the medium and high dosages with comparison to the control group (*P* < 0.01). However, there were no significant differences in the pharmacological actions between the three* Elaeagnus* leaves. It implies that* E. henryi* and* E. lanceolata *had good pharmacological effects on therelaxant, antitussive, and expectorant activities as well as* E. pungens*.

*E. pungens* leaf is acclaimed to treat asthma and chronic bronchitis induced by weakness of lung Qi in the view of the traditional theory of traditional Chinese medicine. In our previous study, chemical components of* Elaeagnus pungens* leaf are isolated, purified, and identified. The results indicates that it mainly contains many flavonoids of which the chemical structures are characterized by quercetin, kaempferol, and isorhamnetin as aglycones linking with glycosyl groups [18, 19]. Many other researches have verified that flavonoids from Chinese herbs are effective on antiasthmatic, antitussive, and expectorant properties, for example, naringenin from* Exocarpium Citri* Grandis and total flavones from* Acanthopanax senticosus* [26–29]. Moreover, plants from* Elaeagnus* are reported to have flavonoid constituents such as* E. henryi*,* E. lanceolata*,* E. angustifolia, *and* E. bockii* [8–10, 21]. It prompted us to lay a hypothesis that these flavonoids in the three* Elaeagnus* leaves might have the antiasthmatic, antitussive, and expectorant activities. So, we analyzed the chemical components and contrasted flavonoids peaks by HPLC-DAD. The result showed that the three* Elaeagnus* leaves had more similarity on overlap peaks between the 15 and 60 min (Figure 6) and the peaks belonging to flavonoids compounds (Table 1). However, due to the nature of multiple chemical constituents involved in the natural plants as well as the multifactorial condition of asthma, it is very important to further separate chemical constituents from the three* Elaeagnus* leaves being effective on the relief of bronchoconstriction, inhibition of cough, and increase of secretion output. Further studies are necessary to clarify the mechanism by which the three* Elaeagnus* leaves possess the antiasthmatic, antitussive, and expectorant activities.

In conclusion, our study indicated that the water extracts of* Elaeagnus pungens*,* Elaeagnus henryi,* and* Elaeagnus lanceolata *leaf demonstrated the significantly antiasthmatic, antitussive, and expectorant effects* in vivo*. These effects were the important evidence for the traditional use of* E. henryi* leaf and* E. lanceolata *leaf as antiasthmatic remedy. Moreover, there was more similarity on overlap peaks between the 10 and 40 min retention time by HPLC and the peaks belonging to flavonoids compounds, suggesting that the main constituents of the three* Elaeagnus *leaves were flavonoid for the pharmacological activities. It persuaded us to draw a conclusion that* E. henryi* and* E. lanceolata *played an important role as well as* E. pungens *to treat asthma and chronic bronchitis.

## Figures and Tables

**Table 1:** Flavonoids peaks of *E. pungens*,* E. lanceolata,* and *E. henryi*. leaf by HPLC-DAD.

Samples	Retention time/min	UV *λ*_max_/nm
*E. pungens*	10.780	265, 350
	20.650	264, 347
	20.960	264, 346
	23.117	264, 347
	23.747	264, 347
	26.243	264, 344
	27.997	264, 348
	38.773	266, 313
	39.917	266, 308

*E. lanceolata*	18.520	255, 353
	20.960	262, 350
	23.777	264, 347
	25.770	240, 318
	26.260	264, 344
	28.020	264, 350
	38.797	266, 313
	39.940	266, 313

*E. henryi*	15.323	237, 327
	17.160	238, 327
	18.240	255, 352
	20.683	264, 346
	20.997	264, 342
	21.643	255, 355
	23.157	264, 350
	23.897	242, 333
	28.063	264, 355
	38.817	266, 313
	39.963	266, 315
	41.800	240, 325
